# General Acceptance of Antiseptic Methods

**Published:** 1882-02

**Authors:** 


### GENERAL ACCEPTANCE OF ANTISEPTIC METHODS.

In speaking of the open treatment of wounds, I have touched on the opposition which the new doctrine at first met with on so many sides. You all know well how violent this was. Even in science great changes cannot be brought about without injury to numerous individual interests; but I insist that, nowadays, no serious opposition exists; that there is no surgeon who would dare, at the operating-table, or in the treatment of the wounded, quite to renounce antiseptic methods; who calmly and with a good conscience would tread the paths which, fifteen years ago we all followed like sheep. Even the most obstinate have had to give way to the great principles of the new era, and often in a much higher degree than they acknowledge to themselves or others---the necessity not only of the disinfection of hands, sponges, instruments, and bandages, but also the necessity of a primary disinfection of fresh wounds, is, I believe, universally acknowledged.---*Volkmann, Int. Med. Congress.*

